# The effects of protein dietary supplementation on fecal egg counts and hematological parameters in goat kids with subclinical nematodosis

**DOI:** 10.14202/vetworld.2015.1351-1355

**Published:** 2015-11-24

**Authors:** Priyanka Konwar, S. P. Tiwari, M. Gohain, Kiran Kumari

**Affiliations:** Department of Animal Nutrition, College of Veterinary Science & Animal House, Chhattisgarh Kamdhenu Vishwavidhyalaya, Durg, Chhattisgarh, India

**Keywords:** fecal egg count, goat kids, hematological parameter, nematodosis, protein dietary supplementation

## Abstract

**Aim:**

The aim of the present study was to assess the effect of dietary supplementation with different levels of protein on fecal egg counts and hematological parameters in goat kids with subclinical nematodosis under semi-intensive condition.

**Materials and Methods:**

20 goat kids (3-5 months old with an average body weight of 8.90 kg) were randomly allocated to four groups: T1, served as a negative control, without receiving concentrate feed, and T2, T3, and T4 that received concentrate feed containing 16, 20, and 24% digestible crude protein, respectively. The experiment was carried out for 60 days.

**Results:**

In this study, protein supplementation had a significant (p<0.05) effect on fecal egg counts even after 15 days; hemoglobin (Hb) (g/dl) after 45 days; total leukocyte count (10^3^/mm^3^) and total erythrocyte count (10^6^/mm^3^) after 30 days; packed cell volume (%), lymphocyte (%), and eosinophil (%) after 15 days of supplementation, whereas monocyte (%) and neutrophil (%) values were not significantly influenced by protein supplementation effect during the entire experiment. The values of mean corpuscular volume (fl) were affected significantly (p<0.05, p<0.01) due to protein supplementation after 30 days, mean corpuscular Hb (MCH) (pg) after 45 days, but MCH concentration (g/dl) was not significantly different among the experimental groups during the entire experiment.

**Conclusion:**

The dietary supplementation with different levels of protein significantly improved the hematological profiles and inhibited the nematodosis infection in the experimental goat kids.

## Introduction

Gastrointestinal parasitism is a major problem in a small ruminant production worldwide, due to its impact on animal health and productivity and the associated costs of control measures [1]. The usual strategy of gastrointestinal nematodes control based on the repeated use of anthelmintics and is nowadays under question because of the increasing development of resistance to these molecules [2].

The manipulation of host nutrition is the alternative method to anthelmintics and is intended to improve the host resistance and/or resilience against parasitic infections. Research has already shown that increased dietary intake of metabolizable protein and energy and combined with high-quality pasture can directly promote the host resistance and resilience against worm infection by maintaining tissue and/or blood homeostasis and production [3,4]. An improvement of the host diet contributes to maintain the tissue and/or blood homeostasis and the host production despite the presence of worms.

The present study was, therefore, carried out to study the effect of protein dietary supplementation on Egg per gram (EPG of feces) and different hematological parameters in goat kids infected with natural subclinical nematodosis.

## Materials and Methods

### Ethical approval

Permission of the Institutional Animal Ethics Committee was taken prior to the start of the experiment.

### Experimental animal, diet, design, and management

20 indigenous goat kids of 3-5 months old infected by natural subclinical nematodosis were selected for the experiment conducted in the Department of Animal Nutrition, College of Veterinary Science and Animal Husbandry, Anjora, Durg (Chhattisgarh) and randomly allocated to four groups of five animals each (T1 served as a negative control, without receiving concentrate feed, and T2, T3, and T4 that received concentrate feed containing 16%, 20%, and 24% digestible crude protein [DCP], respectively). They were ear tagged for identification and experiment was carried out for 60 days with the completely randomized design under semi-intensive condition. Animals were individually fed and were reared under same conditions of hygiene and management.

The animals grazed in a pasture containing mixed grass predominant with sola grass (*Aeschynomene indica*) for 4 h (8 am to 12 noon) and concentrate feed (100 g/day/animal) was offered at 3:30 pm. Fecal egg count was were determined to confirm the occurrence of nematodosis. Diets were formed by using crushed maize, soybean meal, de-oiled rice bran; in detail, T2, T3, and T4 diets contained 70% of total digestible nutrients and 16%, 20% and 24% of DCP, respectively. The diets were further supplemented with mineral mixture (2%) and salt (1%). The diets were formulated as per standard requirement [5].

### Blood and fecal sample collection

The fresh fecal samples from all kids were individually collected using hand gloves at 0, 15, 30, 45, and 60 days. The samples were put in the plastic bags for egg counting as described by the modified McMaster techniques [6]. Blood samples were also collected from all kids at 0, 15, 30, 45 and 60 days. In detail, 2 ml blood was taken in a glass vial containing ethylene diamine tetra acetate appropriate for hematological analyses. Immediately after blood collection, the tubes were gently rotated between palms to mix it with anticoagulant. Hemoglobin (Hb), packed cell volume (PCV), total erythrocyte count (TEC), total lymphocyte count (TLC), differential leukocyte count - monocyte, lymphocyte, neutrophil and eosinophil, mean corpuscular volume (MCV), mean corpuscular Hb (MCH), and MCH concentration (MCHC) were performed as described by Jain [7].

### Statistical analysis

The results obtained during this study were analyzed by as per Snedecor and Cochran [8] using software package SPSS version 16.0 [9].

## Results and Discussion

### EPG

The mean values of EPG (Table-1) significantly differ (p<0.05) among the treatments. The highest values were obtained in T1 and lowest in T3 and T4, even from the first 15 days of protein dietary supplementation. The values were increased throughout the experiment possibly due to flock grazing. Especially, T3 have significantly (p<0.01) lower values than T1 and T2 groups suggesting that the supplementation influenced the rate of larval development in the kids. It might also be an effect of resistance and/or resilience increase against nematodes due to higher protein supplementation level. Previous studies have also revealed that there is a significant reduction in the fecal egg count and worm burden in goats after supplementation with dietary protein [10,11].

**Table-1:** The effect of protein supplementation on fecal egg count in goats.

Parameters	Period (day)	Treatments				Significance

		T1	T2	T3	T4	
EPG	0	1520±73.48	1480±73.48	1520±37.42	1500±70.71	NS
	15	1860±50.99^b^	1720±58.31^ab^	1680±37.42^a^	1620±73.48^a^	*
	30	2240±67.82^b^	1920±58.31^a^	1820±37.42^a^	1840±81.24^a^	**
	45	2580±48.99^b^	2080±58.31^a^	1920±37.42^a^	2020±96.95^a^	**
	60	2920±58.31^c^	2320±58.31^b^	2040±50.99^a^	2220±86.02^ab^	**

abc Means within a row with different superscripts are significantly different

(* p<0.05,

** p<0.01).

NS=Not significant

### Percentile depiction of nematode genera

From the pooled coproculture, the infective larvae were harvested, and it was observed that predominantly *Haemonchus* sp. were present in all the treatments at a ratio of 9:1.

### Hematological profiles

The Hb concentration significantly differed (p<0.01) amongst the different groups at 45^th^ and 60^th^ day of the experiment (Table-2). The concentration at the 45^th^ day was higher in protein supplemented groups and at the 60^th^ day, values were the highest in T3 and T4. The concentration of Hb decreased as the levels of infection and incubation period increased. However as the level of protein supplementation increased, the Hb content also increased after the 30^th^ day of the experiment, especially after the 45^th^ day. In the present study, a reduction in Hb content was observed, possibly as a result of either blood loss due to blood-sucking by larvae and adults [12] or due to mucosa damage [13]. Al-Rekani [14] revealed that there is significant reduction in Hb in infected goats compared to control. With increase in parasitemia rates, Hb concentration significantly decreased in small ruminants naturally infected with parasites [15]. Parasitic anemia is related with low Hb levels, eosinophilia and a moderate lymphopenia in naturally infected sheep [16]. Abdel Hameed *et al*. [17] reported higher values of Hb in diets with a higher level of protein supplementation in Sudan desert lambs compared to other experimental diets.

**Table-2:** The effect of protein supplementation on hematological parameters in goats.

Parameters	Period (days)	Treatments				Significance

		T1	T2	T3	T4	
Hb (g/dl)	0	12.58±0.63	11.28±0.31	11.32±0.27	11.44±0.27	NS
	15	10.56±0.70	10.38±0.33	10.46±0.20	10.64±0.27	NS
	30	8.82±0.49	9.36±0.26	9.80±0.19	9.88±0.26	NS
	45	7.08±0.39^a^	8.52±0.38^b^	9.24±0.17^b^	9.10±0.23^b^	**
	60	5.72±0.25^a^	7.18±0.27^b^	8.66±0.16^c^	8.30±0.26^c^	**
TLC (10^3^/mm^3^)	0	7.14±0.11	7.17±0.16	7.18±0.11	7.27±0.16	NS
	15	7.25±0.12	7.37±0.17	7.56±0.12	7.59±0.17	NS
	30	7.39±0.09^a^	7.61±0.17^ab^	7.98±0.12^b^	7.92±0.16^b^	*
	45	7.51±0.07^a^	7.83±0.18^ab^	8.39±0.12^c^	8.22±0.16^bc^	**
	60	7.65±0.06^a^	8.03±0.19^a^	8.80±0.12^b^	8.51±0.15^b^	**
TEC (10^6^/mm^3^)	0	12.34±0.14	12.48±0.18	12.36±0.15	12.42±0.19	NS
	15	11.71±0.15	12.04±0.18	12.09±0.14	12.06±0.20	NS
	30	11.04±0.16^a^	11.58±0.18^b^	11.74±0.19^b^	11.66±0.19^b^	*
	45	10.35±0.15^a^	11.08±0.17^b^	11.49±0.12^b^	11.26±0.20^b^	**
	60	9.65±0.16^a^	10.56±0.19^b^	11.19±0.14^c^	10.85±0.20^bc^	**
PCV (%)	0	33.26±0.35	32.74±0.30	32.92±0.40	32.66±0.38	NS
	15	29.38±0.66^a^	29.86±0.21^a^	31.90±0.35^b^	30.42±0.47^ab^	**
	30	25.52±0.71^a^	26.68±0.26^ab^	30.58±0.35^c^	28.40±0.44^b^	**
	45	21.48±0.74^a^	23.58±0.23^b^	29.04±0.31^d^	26.70±0.44^c^	**
	60	17.88±0.76^a^	20.02±0.37^b^	27.52±0.21^d^	24.26±0.46^c^	**

abc Means within a row with different superscripts are significantly different

(* p<0.05,

** p<0.01).

Hb=Hemoglobin, PCV=Packed cell volume, TEC=Total erythrocyte count, TLC=Total leukocyte count, NS=Not significant

The mean TLC values significantly differed among the experimental groups on day 30 (p<0.05) and on days 45 and 60 (p<0.01) of the experiment; in general, T3 and T4 had higher values compared to T1 and T2 group (Table-2). TLC value increased as the levels of infection and incubation period increased, possibly as a result of parasitic infection. Qamar [18] reported that TLC values were significantly affected by the extent of nematode parasitosis. In the case of parasitized animals that are supplemented with proteins, the number of eosinophils and other leukocytes, mast cells and proteases released from the mast cells increased in the mucosa of the gastrointestinal tract [19]. TLC showed a significantly increased value in small ruminants naturally infected with parasites [15].

The mean values of TEC significantly differed (p<0.05, p<0.01) among the experimental groups during the experiment apart from the day 0 to 15 (Table-2). Lower values were shown in T1 than the other treatments. Decreased level of TEC as a result of increase in parasitemia were reported in small ruminants with natural parasitic infestation [14,15]. Abdel Hameed *et al*. [17] reported higher TEC values in diets supplemented with different level of protein in Sudan desert lambs compared to other experimental diets.

The mean PCV values significantly differed (p<0.01) among the experimental groups during the entire period apart from day 0 (Table-2). On day 30, 45, and 60, T3 showed the highest and T1^th^e lowest values compared to T2 and T4 group, possibly as a result of the improved nutrition of the parasitized animals. The higher dietary protein improved the erythropoiesis or reduced the establishment or development of adult nematodes. The kids on the higher protein diet even with heavy infection better able to resist the adverse effect of gastrointestinal nematodosis. Al-Rekani [14], Esmaeilnejad [15] also reported decreased values of PCV in infected compared to control animals.

The influence of protein supplementation level on monocyte and neutrophil values are also presented in Table-3. No significant differences were found among the treatments. The values of monocytes did not follow any specific trend and in the case of neutrophil; there was a decreasing but not significant trend in T2, T3, and T4. Esmaeilnejad *et al*. [15] reported that neutrophil counts were significantly increased with the increase of parasitemia rates in natural parasitic small ruminants, a finding that is in accordance with the present study.

**Table-3:** The effect of protein supplementation on DLCs in goats.

Parameters	Period (days)	Treatments				Significance

		T1	T2	T3	T4	
Monocytes (%)	0	1.60±0.75	1.80±0.80	1.40±0.98	1.40±0.98	NS
	15	1.40±0.60	1.40±0.98	1.40±0.87	1.20±0.97	NS
	30	1.60±0.81	1.20±0.97	1.40±1.40	1.40±0.98	NS
	45	1.20±0.73	1.20±1.20	1.60±1.03	1.60±1.17	NS
	60	2.00±0.89	1.20±0.97	1.40±1.40	1.40±1.17	NS
Lymphocytes (%)	0	51.60±0.93	51.40±0.93	51.60±1.03	51.60±0.75	NS
	15	50.80±0.80^d^	48.00±0.55^c^	44.40±0.60^a^	46.20±0.37^b^	**
	30	48.80±0.37^d^	46.20±0.37^c^	42.00±0.45^a^	44.20±0.37^b^	**
	45	45.80±0.58^d^	44.20±0.37^c^	40.20±0.37^a^	42.00±0.32^b^	**
	60	43.20±0.58^c^	42.20±0.37^c^	38.20±0.37^a^	39.80±0.37^b^	**
Neutrophils (%)	0	41.80±0.80	42.00±0.55	41.80±0.80	41.60±0.75	NS
	15	42.00±0.84	41.00±0.89	41.00±1.05	41.00±0.89	NS
	30	42.00±0.84	40.00±0.71	40.40±0.75	40.20±0.66	NS
	45	42.20±1.02	39.00±0.89	39.80±1.07	39.40±0.93	NS
	60	42.20±1.02	38.40±0.93	39.40±0.93	38.60±0.81	NS
Eosinophils (%)	0	5.20±0.37	5.40±0.24	5.00±0.32	5.20±0.20	NS
	15	9.20±0.37^b^	8.40±0.24^b^	7.00±0.32^a^	7.40±0.24^a^	**
	30	13.20±0.37^d^	11.40±0.24^c^	8.20±0.37^a^	9.40±0.24^b^	**
	45	16.20±0.37^d^	13.80±0.49^c^	9.40±0.24^a^	11.40±0.24^b^	**
	60	19.20±0.37^d^	16.40±0.24^c^	10.80±0.20^a^	13.60±0.24^b^	**

abc Means within a row with different superscripts are significantly different

(** p<0.01). NS=Not significant, DLC=Differential leukocyte count

The influence of protein supplementation on lymphocyte count in kids was significant (p<0.01) from the 15^th^ day (Table-3). The highest values were constantly showed by T1 and lowest by T3 group. The results of the present study might be due to the chronic infection of nematodosis resulting in the exhausted immune system. Esmaeilnejad *et al*. [15] reported that number of lymphocyte increased significantly in natural parasitic small ruminants which were in accordance with the present findings.

As presented in Table-3, eosinophil count was significantly influenced by protein supplementation apart from day 0. The eosinophil count was higher in T1 followed by T2, T4 and T3. It has already been suggested that eosinophil count may provide an index of protective immune response of animals to parasitism [20]. With increase in parasitemia rates, eosinophil count shows a significantly increased value in small ruminants with natural parasitic infection [15].

The average values of MCV, MCH, and MCHC of the experimental groups are presented in Table-4. The MCV values were higher in T3 and T4 compared to other groups on days 30, 45 and 60. The MCH values were higher in T3 and T4 compared to T1 group on days 45 and 60. No significant differences in MCHC values were found among the groups. However, the values were decreased as the time period and level of infection increased. The present findings of MCV and MCH were supported by previous studies in goats infected with parasites [15]. However, in contrast with the present study, MCV appeared to slightly increase with increase in worm load in cattle affected by fascioliasis [21]. Abdel Hameed *et al*. [17] also reported higher values in diets with different level of protein supplementation in Sudan desert lambs compared to other experimental diets which were in accordance with present findings. MCHC showed no reasonable change with increase in worm load in cattle affected with fascioliasis [21]. Reduced level of MCHC with the increase in infection was also observed in small ruminants with natural parasitic infection [15]. At the same time, higher values of MCHC were recorded in diets with different level of protein supplementation in Sudan desert lambs compared to other experimental diets [17].

**Table-4:** The effect of protein supplementation on red blood cell indices in goats.

Parameters	Period (days)	Treatments				Significance

		T1	T2	T3	T4	
MCV (fl)	0	26.96±0.29	26.24±0.28	26.64±0.55	26.31±0.57	NS
	15	25.08±0.53	24.81±0.29	26.38±0.47	25.25±0.68	NS
	30	23.12±0.59^a^	23.04±0.27^a^	26.08±0.69^b^	24.39±0.64^ab^	**
	45	20.76±0.64^a^	21.29±0.43^a^	25.28±0.47^b^	23.72±0.61^b^	**
	60	18.51±0.67^a^	18.98±0.56^a^	24.61±0.41^c^	22.38±0.62^b^	**
MCH (pg)	0	10.21±0.57	9.05±0.30	9.17±0.32	9.23±0.27	NS
	15	9.03±0.63	8.63±0.31	8.65±0.23	8.83±0.28	NS
	30	8.00±0.46	8.09±0.25	8.35±0.24	8.48±0.28	NS
	45	6.85±0.39^a^	7.70±0.38^ab^	8.04±0.20^b^	8.09±0.27^b^	*
	60	5.94±0.35^a^	6.81±0.32^ab^	7.75±0.21^b^	7.66±0.29^b^	**
MCHC (g/dl)	0	37.83±1.89	34.47±1.04	34.40±0.86	35.03±0.78	NS
	15	35.93±2.16	34.76±1.07	32.80±0.65	34.98±0.70	NS
	30	34.55±1.58	35.09±1.01	32.06±0.75	34.79±0.84	NS
	45	33.00±1.60	36.09±1.33	31.84±0.75	34.12±1.04	NS
	60	32.30±2.34	35.82±0.75	31.48±0.76	34.27±1.33	NS

abc Means within a row with different superscripts are significantly different

(* p<0.05,

** p<0.01).

NS=Not significant, MCV=Mean corpuscular volume, MCH=Mean corpuscular hemoglobin, MCHC=Mean cell hemoglobin concentration

## Conclusion

It may be concluded that dietary supplementation with different levels of protein in goats with subclinical nematodosis had significant beneficial effects on reducing the worm egg count and on improving hematological profile with respect to Hb, TLC, TEC, PCV, lymphocyte, eosinophil, MCV, and MCH. Thus, protein-rich concentrate feed may be used as part of a sustainable strategy to control gastrointestinal nematodosis in goat kids under semi-intensive conditions.

## Authors’ Contributions

PK carried out the experiment. SPT - Designed and guided the experiment. MG and KK help in sample processing and data analysis. All authors read and approved the final manuscript.
